# Face Recognition on a Smart Image Sensor Using Local Gradients

**DOI:** 10.3390/s21092901

**Published:** 2021-04-21

**Authors:** Wladimir Valenzuela, Javier E. Soto, Payman Zarkesh-Ha, Miguel Figueroa

**Affiliations:** 1 Department of Electrical Engineering, Universidad de Concepción, Concepción 4070386, Chile; wladivalenzuela@udec.cl (W.V.); javsoto@udec.cl (J.E.S.); 2 Department of Electrical and Computer Engineering (ECE), University of New Mexico, Albuquerque, NM 87131-1070, USA; pzarkesh@unm.edu

**Keywords:** smart image sensor, smart pixel, vision chip, intelligent sensor, feature extraction, face recognition, linear binary patterns, linear discriminant analysis, field-programmable gate array, very large-scale integration

## Abstract

In this paper, we present the architecture of a smart imaging sensor (SIS) for face recognition, based on a custom-design smart pixel capable of computing local spatial gradients in the analog domain, and a digital coprocessor that performs image classification. The SIS uses spatial gradients to compute a lightweight version of local binary patterns (LBP), which we term ringed LBP (RLBP). Our face recognition method, which is based on Ahonen’s algorithm, operates in three stages: (1) it extracts local image features using RLBP, (2) it computes a feature vector using RLBP histograms, (3) it projects the vector onto a subspace that maximizes class separation and classifies the image using a nearest neighbor criterion. We designed the smart pixel using the TSMC 0.35 μm mixed-signal CMOS process, and evaluated its performance using postlayout parasitic extraction. We also designed and implemented the digital coprocessor on a Xilinx XC7Z020 field-programmable gate array. The smart pixel achieves a fill factor of 34% on the 0.35 μm process and 76% on a 0.18 μm process with 32 μm × 32 μm pixels. The pixel array operates at up to 556 frames per second. The digital coprocessor achieves 96.5% classification accuracy on a database of infrared face images, can classify a 150×80-pixel image in 94 μs, and consumes 71 mW of power.

## 1. Introduction

The attention of the scientific and industrial community in image-based biometric methods has fostered a growing interest in smart imaging systems (SIS) that can handle the computational requirements of real-time video analysis. Biometrics is described as a pattern-recognition technique for individual identification, based on their physical, chemical, or behavioral characteristics [1,2]. One of the most popular biometric techniques is face recognition [2], which has abundant applications [3,4,5] in various areas, such as: (1) security, including identity verification [6,7], computer or mobile device unlock [7,8], criminal records search, and voter registration; (2) surveillance, such as cameras used on closed circuit television (CCTV) [9]; and (3) access control that could grant access to a specific place or an electronic account to a group of people [10] using their faces as a credential. As described by Das et al. [11], there is a growing attention from the scientific community on mobile devices with biometric recognition. This attention is mainly fueled by the commercial interest in robust authentication methods for smartphones, laptops, tablets, and other mobile devices [7,8].

Different low-power biometric sensors have been reported in the literature, such as adaptive wireless body sensor networks for biometrics and healthcare applications [12] for long-time monitoring, sensors for age and gender classification which monitor brain signals using electroencephalography (EEG) [13], biometric recognition systems for mobile Internet-of-things (IoT) devices [14], and an ultra-low-power hybrid face recognition processor integrated with a CMOS image sensor (CIS) [15] applied to mobile devices [16,17]. Common to all these designs are two technological challenges: low power consumption and circuit area reduction. Both are intimately related to key features of mobile devices, such as energy autonomy and size [12].

Although face recognition is a popular method of security and authentication, it is vulnerable to presentation attacks (PAs), especially when the face images are acquired in the visible spectrum. PAs are different techniques and methods that intruders use to infringe and deceive face recognition systems, such as presenting the system with an authorized face image using a 3D silicon mask [18] or a 2D image on a printed photograph or even a mobile device screen [19]. Modern devices implement presentation attack detection (PAD) methods using complementary information from near-infrared (NIR) [20] or thermal [21] image sensors, or using deep convolutional neural network methods for anomaly detection [22]. Infrared (IR) face recognition is particularly attractive [23,24] because it has shown to be more robust against variations in global illumination than using images in the visible spectrum, and because thermal IR face images are also more difficult to forge [25]. Therefore, implementing fast and robust face recognition algorithms in resource- and power-constrained portable and mobile devices is an important area of research [19,26].

To reduce power consumption and increase hardware integration, designers often turn to dedicated hardware architectures specifically designed to perform a singular task of interest. In image processing, there is visible progress in the development of smart imaging sensors (SIS), also referred to as vision chips. The SIS are dedicated electronic devices that combine conventional image sensors with additional circuitry on the same die [27]. The additional circuitry performs, either partially or totally, operations and algorithms associated with different image processing methods. We can organize image processing methods and hardware into three levels, depending on where the data is processed: pixel level processing, column/row level processing, and data-sequence level processing, i.e., processing that occurs outside the pixel array after digital conversion [27]. Of these three levels, the most challenging when designing the architecture of a SIS is the pixel level. This is because there is a limited area available inside each of the pixels, and the design of the processing circuits must minimize the overhead imposed on the pixel size. Therefore, the complexity of the circuitry is limited by the space that can be occupied and, as a consequence, the complexity of the image-processing operations that can be introduced into the pixel is also limited. This difficult tradeoff can be observed on the SIS work available the literature. Some examples of image processing methods implemented as a SIS are edge detection [28,29,30], image classification using analog lightweight convolutional neural networks [31], on-chip nonuniformity compensation on IR image sensors [32,33], target tracking [28,30], motion detection [28], feature extraction [34,35,36,37,38], and face recognition [15,16,17], among others [39].

When a pixel sensor performs a significant level of data processing, such as complex mathematical operations or feature extraction, then it is frequently referred to as a smart pixel. Smart pixels have the capacity to deliver a high level of fine-grained parallelism, where each smart pixel of the focal plane array (FPA) in a SIS performs computation simultaneously on different data [27]. Fine-grained parallelism can improve the execution time, lower the latency, reduce the amount of memory required to store temporary results, and maximize throughput [36,37]. Moreover, when the smart pixel operates in the analog domain, they can also reduce power consumption and die area [40].

In previous work, we proposed an intelligent readout integrated circuit (iROIC) that computes local gradients on-chip [41]. This iROIC includes the architecture of an analog smart pixel that can be programmed to compute the local differences between neighbor pixels during integration time. We showed the design and circuit-level simulations of a smart pixel that computes the local gradients, and proposed a face recognition algorithm, implemented in software, that uses local gradients to extract features from the input image.

In this paper, we present the design and evaluation of a complete SIS architecture based on the algorithm and smart-pixel design in [41] to perform face recognition during image capture. The proposed SIS design is suitable for mobile devices, where the SIS can be operated as a conventional sensor to capture images of a scene, or as a face recognition system to obtain the identity of a subject. The smart pixel is based on a capacitive transimpedance amplifier (CTIA) integrator, which is widely used in thermal IR imagers, but our face recognition method can be used in visible and NIR images as well. Our smart pixel adds a small number of transistors to the CTIA integrator, thereby minimizing the added cost in area and power compared to a regular image sensor.

Our results show that it is possible to use analog pixel-level processing with a minimal penalty on fill factor, and obtain results that are comparable to a fully digital implementation of the algorithm. The heterogeneous SIS architecture is composed of two processing stages, in the analog and digital domains. The analog stage is a bidimensional smart-pixel array, which can be configured to capture a scene as a conventional CIS or to extract the features of the captured image during the integration time. The digital stage is composed of the standard circuitry for readout, and a digital coprocessor that uses the extracted features to compute facial recognition. We validated the complete design of the SIS, simulating the **analog smart-pixel array after parasitic extraction of the complete circuit layout using the** TMSC 0.35 μm process. We also designed and implemented the digital coprocessor on a Xilinx XC7X020 field-programmable gate array (FPGA). We tested the performance of the complete SIS using a database of faces in the thermal IR spectrum, which consists of 605 images of 53 different individuals. In the 0.35 μm TSMC process, the smart-pixel circuit measures 30 μm × 22.5 μm. Without the image processing circuitry, the base design of a 32 μm × 32 μm pixel achieves a fill factor of 47.6%, while adding all our local gradient computation circuits reduces the fill factor to 34%. Moreover, when porting the design to the TMSC 0.18 μm process, the smart pixel achieves a fill factor of 76%. Using an array of 151×80 pixels, the SIS acquires and computes local gradients at 556 frames per second. The digital coprocessor classifies a face image in 94 μs with 96.5% accuracy, compared to 98.5% for a floating-point software version of the algorithm that uses linear binary patterns (LBP), and consumes 71 mW.

The rest of the paper is structured is as follows. In Section 2, we discuss related work. In Section 3, we describe the face recognition method used in the SIS. In Section 4, we describe the proposed SIS architecture, including the smart pixel and the coprocessor and digital controller. In Section 5, we present our performance and classification results. Finally, in Section 6, we conclude with a discussion of the results and possible outcomes related to our work.

## 2. Related Work

The technological advances in high-performance computing have enabled the development of fast and highly accurate biometric systems. Most frequently, this performance is achieved using power-hungry processors and graphics processing units (GPUs) [42,43]. While this cost in power and space may not be important in big data applications that require high precision, it is normally not acceptable in mobile or portable biometric systems [11], which require compact, power-efficient electronics.

In the particular case of facial recognition, researchers have developed special-purpose systems focused on speed, portability, and low power consumption. In the case of fully digital systems, FPGAs are popular implementation platforms because of their high level of fine-grained parallelism and low power consumption, compared to traditional programmable solutions. Šušteršič et al. [44] show a face recognition algorithm based on the fast Fourier transform (FFT) and implemented on a Spartan-3E FPGA, which reaches an accuracy of 79%. Bonny et al. [45] implemented a histogram-based face recognition method on a Zynq-7000 FPGA using 320×243-pixel images, with less than 20% resource utilization and a throughput of one face identification per second with a 100 MHz clock. Ahmed et al. [46] proposed a neural network (NN) classifier using features based on a histogram of oriented gradients (HOG). They implemented the algorithm on a Xilinx Virtex-7 FPGA with a 157 MHz clock, and they report 90% accuracy with 27×18-pixel images at native video frame rate. Qu et al. [47] proposed a convolutional neural network (CNN) for face recognition implemented on a PGT-180H FPGA with a 50 MHz. The circuit reaches 99.25% accuracy with 540×480-pixel images at 400 frames per second. The architecture proposed by Mahale et al. [48] implements a combination of weighted modular principal component analysis (WMPCA) and a radial basis function neural network (RBFNN). It uses a Virtex-6 LX550T FPGA to process 450 128×128 pixel images per second. Soto et al. [49] proposed an embedded face classification circuit for IR images on an FPGA that uses LBP and linear discriminant analysis (LDA), achieves 98.6% accuracy using a thermal IR database of 53 subjects, and can classify 8230 images per second with a power consumption of 309 mW.

FPGA-based solutions require a dedicated interface to communicate the FPGA and the CIS. This requires additional resources, consumes extra power, and, because pixel values are read serially, it limits the parallelism that can be exploited by the algorithm. An alternative to optimize power and performance is to include custom processing hardware on the image sensor [50]. A SIS combines highly parallel analog computation and logic circuits in a single die to execute part of the face recognition algorithm on the image sensor, improving area and power efficiency when compared to programmable or FPGA-based solutions [27], and achieving similar performance. Kim et al. [17] integrates a CIS and a hybrid analog–digital CNN on a single chip to perform face detection and recognition. Performing the first layer of the CNN in the analog domain eliminates the need of an analog-to-digital converter (ADC), which consumes more than half of the power in a conventional CIS [51]. Using analog circuits to perform some of the operations of the algorithm reduces power by 15.7% with a 1.3% reduction in accuracy. Jin et al. [29] proposed a SIS with built-in mask circuits that can be programmed for edge detection. The SIS implements part of the edge detection computation at the column level, integrating static memory (SRAM) and additional circuitry to compare the pixels of two adjacent columns. Limiting the comparison to horizontally neighboring pixels reduces the amount of computation and enables parallelism at the column level. They demonstrate that the SIS can be configured to capture either normal 8-bit images or image edges, with a power consumption of 9.4 mW at 60 frames per second (fps). Zhong et al. [35] describes a SIS with a multimode 128×108-pixel SIS array with omnidirectional LBP and edge detection, which reaches a fill factor of 55% and consumes 12.7 W at 30 fps. Similarly, Gottardi et al. [52] presents a multimode SIS which computes a 3×3-pixel LBP kernel that uses four neighbors. The SIS array dimensions are 64×64 pixels, and it consumes 35 μW at 15 fps with a fill factor of 15%.

The SIS architectures described above use different techniques to integrate computation into the image sensor efficiently, including column-level processing, computing in the analog domain, limiting LBP kernel size, and reducing the number of comparisons in the kernel. These trade-offs mainly aim to reduce computation time and maximize fill factor. It is also important to note that some of the computation can be performed at integration time without waiting for the entire image to be acquired. Indeed, Gottardi et al. [52] computes the difference between neighboring pixels during integration to obtain a simplified version of LBP. In our own previous work [41], we propose analog circuits at the pixel level to compute local spatial gradients during integration, which can be used to perform face recognition using external circuitry.

The literature also shows that infrared face recognition is a good option for enhanced security or PAD [19]. Popa et al. [20] improve PAD performance using a combination of IR and conventional cameras. Hoon et al [26] proposed NIRFaceNet, a variation of the FaceNet method tailored for NIR images. Tested on different NIR data sets, NIRFaceNet achieves accuracies between 73.1% and 94.8%. Hermosilla et al. [53] tested different methods of face recognition on two thermal IR databases, and they achieved their best accuracies using Gabor jet descriptors (96.6%), Weber local descriptors (94.9%), and LBP histograms (92.0%). To the best of our knowledge, none of the smart pixel circuits in the literature have been designed for or tested on IR images. The pixel-level circuit that we present in this paper is based on a CTIA pixel architecture, which is suitable for IR and low-light applications; therefore, our SIS architecture is an attractive solution for IR face recognition in embedded and mobile systems.

## 3. Methods

Typically, object recognition algorithms operate in two stages [3,54]. The first stage extracts features from the image using methods such as LBP [55,56,57], deformable part-based models (DPM) [58,59], or a histogram of oriented gradients (HOG) [60,61,62]. The second stage uses the feature vector to label the image, using classification methods such as nearest neighbors [63,64], support vector machines (SVM) [64,65], or deep neural networks [66,67].

**Figure 1:**
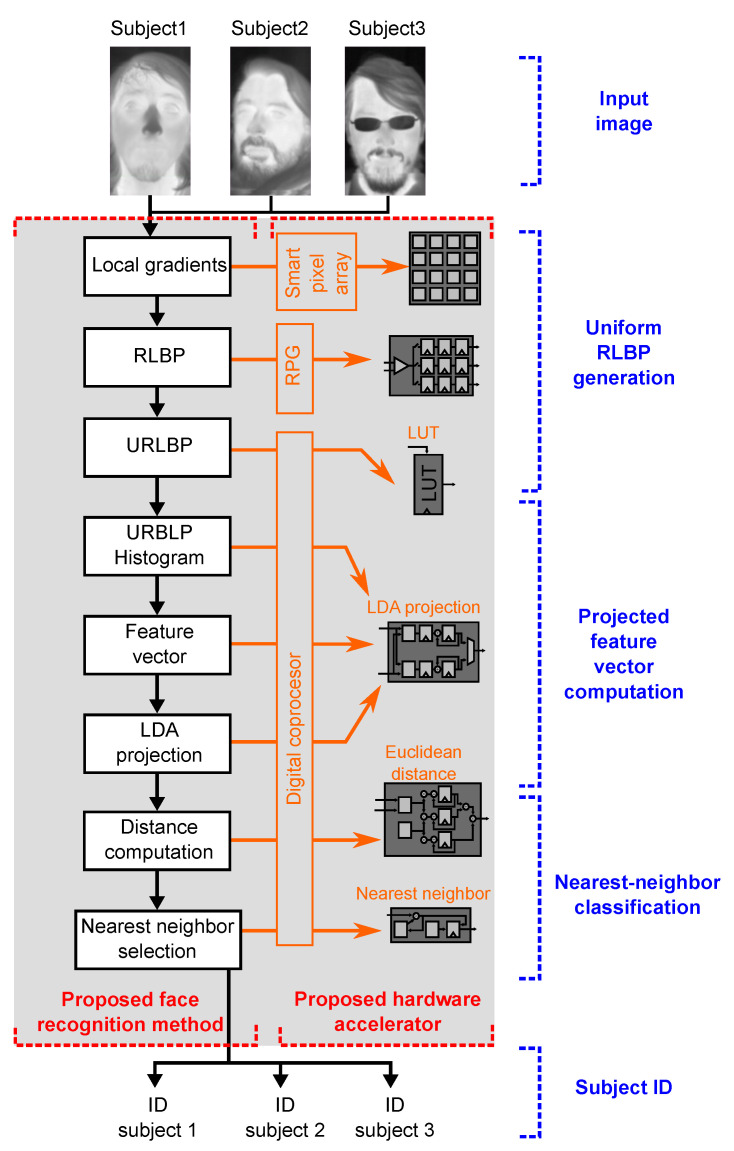
Proposed method and hardware accelerator. The left hand side illustrates the steps of our face recognition algorithm. The right hand side shows the elements of our SIS, namely smart-pixel array, pattern generator, and digital coprocessor, which execute the stages of the algorithm.

Figure 1 depicts a block diagram of the research proposed in this paper. The left hand side of the figure shows the steps of our proposed face classification algorithm, which is described below. The right hand side of the figure relates each step of the algorithm to the component of our proposed SIS that implements it. The architecture of the SIS is described in Section 4.

**Figure 2:**
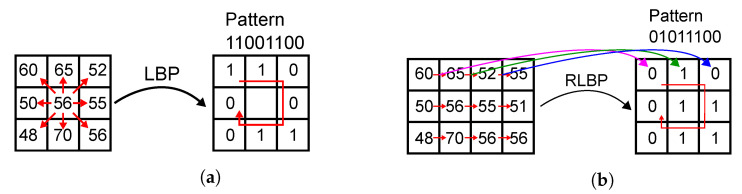
Examples of LBP and RLBP operators on a 3 × 3-pixel window. (**a**) LBP operator with label 11001100. (**b**) RLBP operator with label 01011100.

Algorithm 1 describes our proposed face recognition method, which is based on Ahonen’s LBP-based algorithm [68]. The feature extraction stage replaces LBP with our custom RLBP descriptor and projects the feature vector onto a reduced space using LDA. The classification stage compares the projected vector to a stored database of known faces and selects an ID for the input image using a nearest neighbor criterion. As shown in Algorithm 1, the feature extraction stage first computes an 8-bit RLBP value for each pixel in the image using Algorithm 2. Figure 2 compares regular LBP to RLBP for a 3×3-pixel kernel: LBP, shown in Figure 2a, compares each pixel to its eight neighbors, and concatenates the results to build a binary pattern for the pixel. Thus, LBP requires eight comparisons per pixel. In our RLBP method [41], shown in Figure 2b, each pixel is compared only to its rightmost neighbor and the results from the comparisons of the eight neighbors are concatenated to build the pattern vector. Unlike LBP, RLBP requires only one comparison per pixel, because the result of each comparison is used in eight different kernels. Moreover, all the comparisons in the image can be performed in parallel using only one comparator per pixel. While the features extracted by RLBP contain less information than LBP, the method provides a sufficiently accurate texture representation of the image that achieves a similar performance in face recognition, as shown in Section 5.3.
**Algorithm 1:**Proposed method using RLBP + LDA.
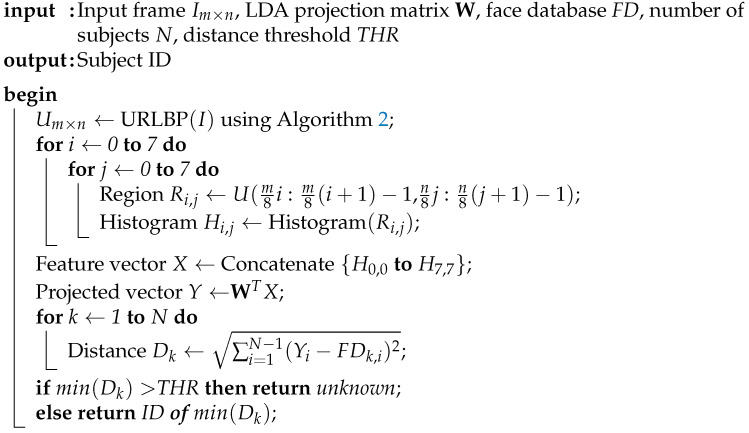

**Algorithm 2:**Uniform RLBP computation.
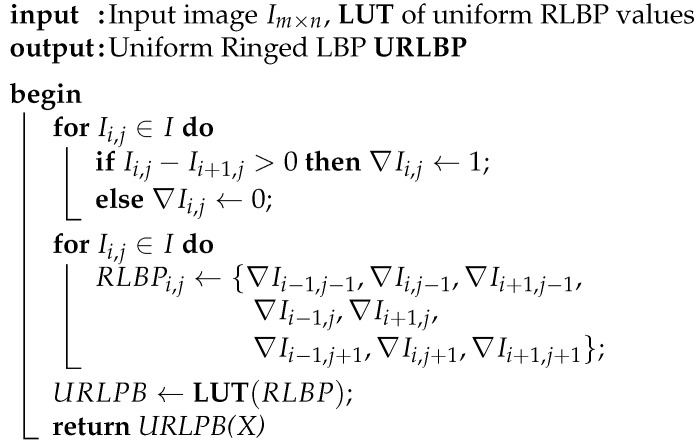



**Figure 3:**
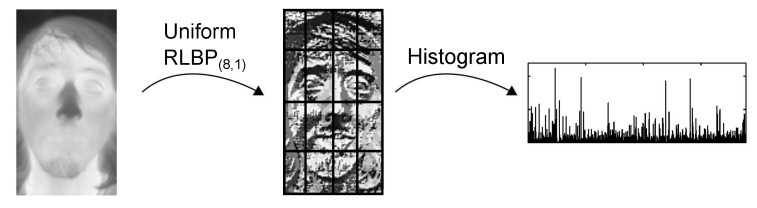
Ahonen’s algorithm using uniform RLBP.

After computing the binary patterns, the algorithm divides the image into 8×8 nonoverlapping regions, and computes a histogram of the binary patterns in each image, as shown in Figure 3. The 64 resulting histograms are concatenated to produce the feature vector that represents the input image. Ahonen [68] uses uniform LBP to reduce the number of labels in the histogram. Uniform LBP assigns a label only to those patterns that have at most two 0-1 or 1-0 transitions between adjacent positions in the 8-bit pattern. As shown in Algorithm 2, we use the same technique, using a 256-entry lookup table (LUT) to map the RLBP values onto uniform RLBP patterns.

After computing the histogram vector, Algorithm 1 uses LDA to map the vector onto a lower-dimensional subspace, as in the Fisherfaces method [69]. LDA applies a linear transformation to the histogram vector, where the transformation matrix **W** is computed to minimize the variance between vectors belonging to the same class (images of the same person) and maximize the variance between vectors of different classes. Using LDA allows us to improve the performance of the classifier, use a simple distance metric, and reduce the dimension of the feature vector, reducing the computational complexity of the classifier. The transformation matrix is computed off-line using a labeled training set, and is used to project the histogram vector *X* onto the new feature space as shown in Equation (1):

(1) $$Y=W^{T}X$$

where *Y* is the linear projection, *X* is the uniform RLBP histogram vector, and *W* is the LDA projection matrix.

Finally, in the classification stage, we compute the Euclidean distance between the projected feature vector and each element of the stored data set *FD* of known subjects. *FD* contains one feature vector for each know face, which is computed as the centroid of all feature vectors obtained from the same subject in the training set. The vectors in *FD* have also been projected using LDA, and the training set can be the same used to compute the LDA matrix **W**. Using the nearest neighbor criterion, we label the input image with the identity of the subject with minimal distance to the projected vector if that distance is larger than a predefined threshold *THR*. If the minimum distance is larger than *THR*, we label the input image as an unknown subject.

## 4. SIS Architecture

**Figure 4:**
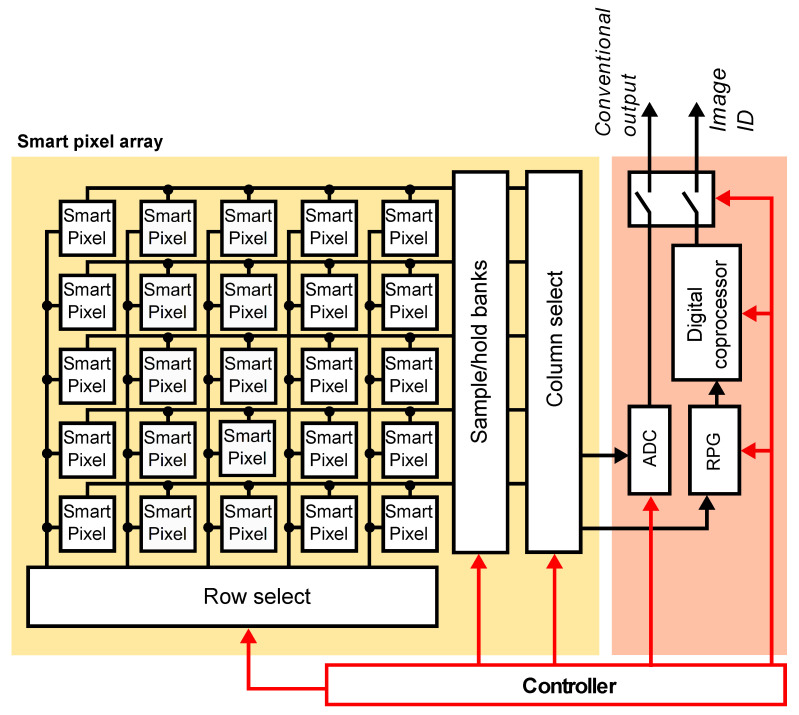
Architecture of the proposed SIS. An array of smart pixels outputs either the pixel value or the difference between horizontally adjacent pixels. An RLBP generator (RPG) reads pixel values and creates an 8-bit RLBP for each pixel in the image. The digital coprocessor computes histograms of RLBP patterns to construct the feature vector, executes the LDA projection on each vector, and selects the nearest neighbor from a stored set of projected vectors using the Euclidean distance.

Figure 4 shows our proposed SIS architecture for face recognition, which can be configured to operate as a conventional image sensor or as a face recognition system. The main components are an array of smart pixels, an RLBP generator (RPG), and a digital coprocessor. The pixel array acquires image data and, in parallel, subtracts the values of horizontally adjacent pixels. The row-select and column-select circuits sequentially read the pixel values and send them to the RPG, which constructs an 8-bit RLBP for each pixel in the image. Dividing the image into 8×8 regions, the digital coprocessor computes a histogram of uniform RLBPs for each region and concatenates them to form the feature vector. Them, it projects the vector using LDA, computes its Euclidean distance to a set of stored vectors corresponding to the known faces, and labels the image using a nearest neighbor criterion.

The SIS can also output a conventional image, in which case each smart pixel outputs the analog voltage output of its readout circuit, and the row- and column-select circuits read the voltages to an ADC that outputs the digital value of the pixels.

### 4.1. Smart Pixel

**Figure 5:**
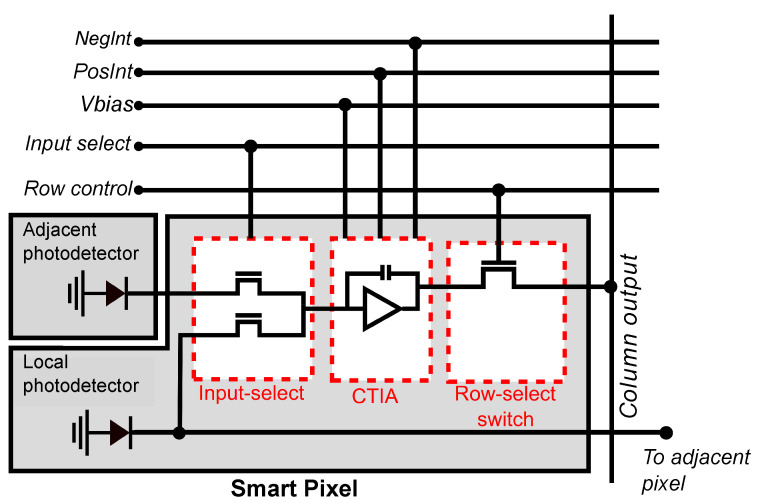
The smart pixel consists of an analog input-select multiplexer, a configurable CTIA, and a row-select switch. All the smart pixels in the array share the control signals placed above in the figure, and all the pixels in the same column share the column output signal. The input to the CTIA can be selected from the photodetector in the local or adjacent pixel.

Figure 5 shows the circuit that implements the smart pixel. It consists of a photodetector, a pair of input-select switches, a programmable CTIA, and a row-select switch. The input of the CTIA are the currents from the local or horizontally-adjacent pixel, selected by the input-select switches. The CTIA computes a voltage that represents either the current pixel value of the difference between adjacent pixels, configured by the global control lines NegInt and PosInt.

Although it uses more area than alternative pixel circuits, using a CTIA for photocurrent integration is a preferred method for low-light environments and IR cameras [70,71,72,73] because its low input impedance offers offers good injection efficiency with weak photodiode currents. In particular, as discussed in Section 5.3, we are interested in using the smart pixel to recognize faces in thermal IR video. Moreover, when compared to other pixel circuits, a CTIA features a wide linear output voltage range [74], small frame-to-frame lag, and reduced noise through better control of the photodiode bias [75].

**Figure 6:**
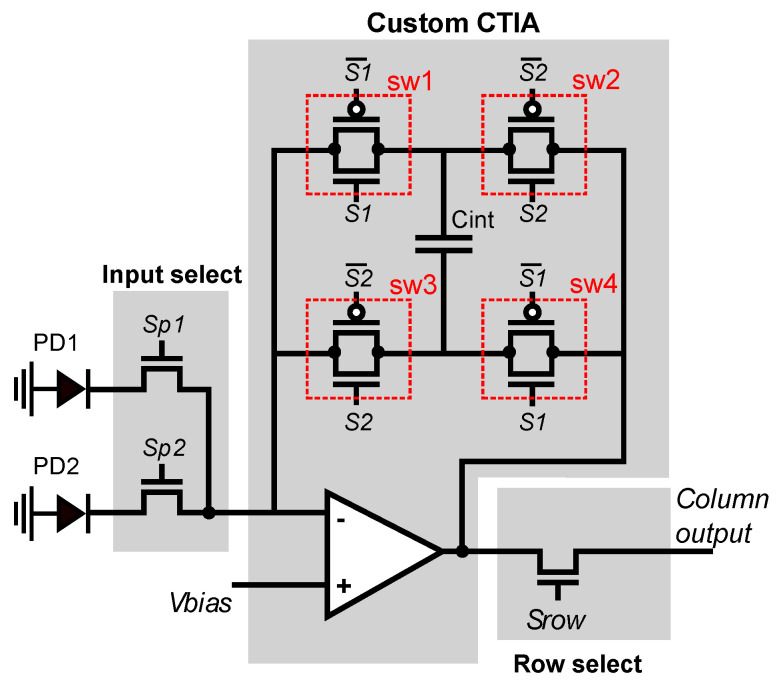
Schematic diagram of the configurable CTIA. The CTIA integrates the photodetector currents and outputs a voltage that represents either the pixel value or the difference between horizontally-adjacent pixels.

Figure 6 shows the schematic of the CTIA. It integrates its input current to produce an output voltage, and a set of four switches, implemented as conventional CMOS transmission gates, can control the orientation of the integration capacitor [76]. The input current comes from the photodetectors in the local or adjacent pixel.

**Figure 7:**
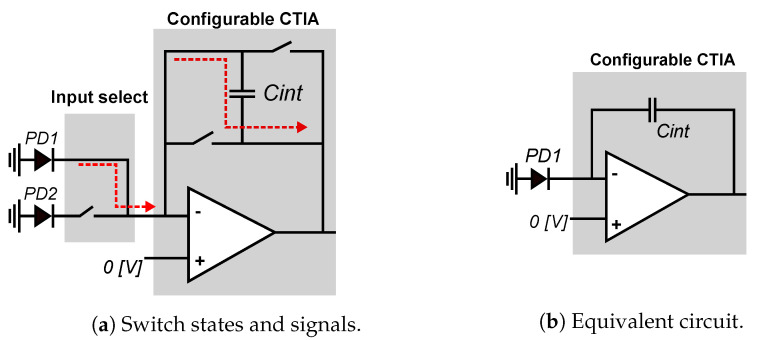
Smart pixel in conventional mode: the input-select switches pass the current from PD1, *sw1* and *sw4* are closed to integrate the current, and *sw2* and *sw3* stay open.

Figure 7a shows the CTIA operating in conventional mode. During the entire integration time, input-control switch connects the CTIA input to the local photodetector PD1. The CTIA is configured in direct mode: *sw1* and *sw4* are closed, and *sw2* and *sw3* are open. The equivalent circuit of Figure 7b shows the CTIA acting as a conventional integrator, and Equation (2) shows how it computes the output voltage that represents the local pixel value:

(2) $$V=I\Delta t/C_{int},$$

where *V* is the output voltage, *I* is the input current from photodetector PD1, Δt is the integration time, and $C_{int}$ is the capacitance value.

**Figure 8:**
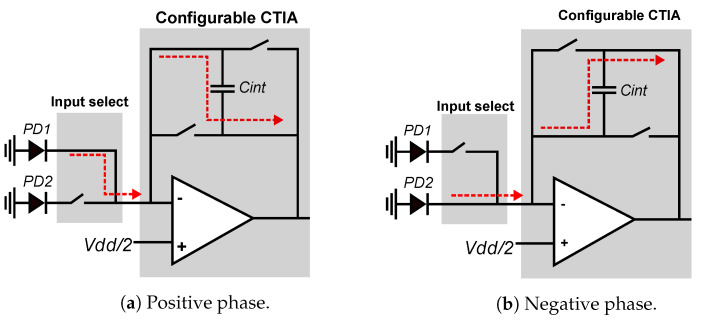
Simplified view of positive and negative integration. During positive integration: *sw1* and *sw4* stay closed, *sw2* and *sw3* stay open. During positive integration: *sw2* and *sw3* stay closed, *sw1* and *sw4* stay open.

Figure 8 shows the smart pixel when configured to compute local horizontal gradients. The global bias input of the CTIA is set to the midpoint between the rails (1.65 V for a 3.3 V supply voltage). The integration time is divided into two phases of equal duration: direct and inverse. During the direct phase, shown in Figure 8a, the CTIA operates in conventional mode by integrating the current from the local PD1 detector, starting from 1.65 V. During the negative phase, shown in Figure 8b, the input switches select the current from the local neighbor pixel PD2, *sw1* and *sw4* are open, and *sw2* and *sw3* are closed. Therefore, during the inverse phase, the CTIA integrates the negative current value of the PD2 photodetector. The output voltage at the end of the integration period is computed as shown in Equation (3):

(3) $$V=\left(I_{1}\Delta t_{s}+I_{2}\Delta t_{s}\right)/\left(2C_{int}\right),$$

where *V* is the output voltage, $I_{1}$ is the input current from the local detector PD1, $I_{2}$ is the current from the adjacent detector PD2, $\Delta t_{s}$ is the integration time, and $C_{int}$ is the capacitance.

In local-gradient mode, the integration time per pixel is reduced by 50% compared to normal operation, which decreases the signal-to-noise ratio. However, this allows us to compute local spatial gradients in parallel on the entire FPA with very small area overhead compared to a conventional integrator. These local gradients are then used by the RPG to compute the RLBP for each pixel.

### 4.2. RLBP Generator

**Figure 9:**
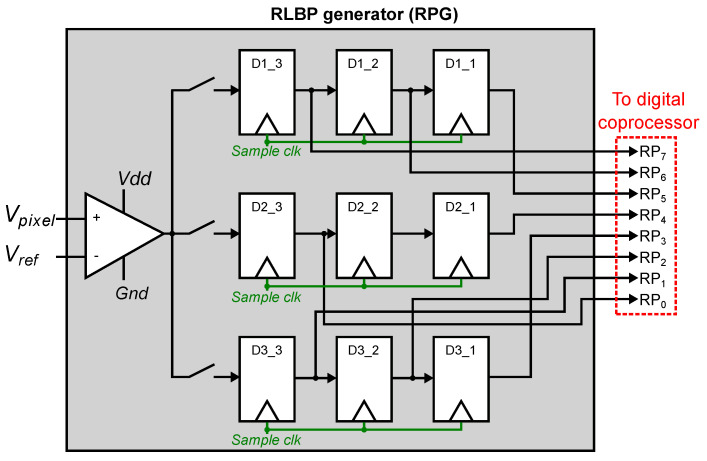
Architecture of the RPG. An input comparator compares the local gradient value for each pixel to a reference voltage. The digital comparator outputs are sequentially stored in an array of 3×3 flip-flops, organized as three shift registers. The RPG outputs an 8-bit RLBP with the output of all the flip-flops except for the one at the center.

Figure 9 shows the topology of the RPG circuit. An input opamp compares the readout value $V_{pixel}$, which represents the difference between two adjacent pixels, to a global reference voltage $V_{ref}$. When $V_{pixel}>V_{ref}$, the digital output of the comparator is 1, and 0 otherwise. The output of the comparator is written into an array of 3×3 flip-flops configured as 3 shift registers, which is used to create the RLBP.

To compute the RLBPs in each region, the RPG performs a row-wise read of the FPA. For each pixel, the RPG reads the pixel value and its two vertically-adjacent neighbors. The comparator output for these values is written into flip-flops D1_3, D3_3 and D3_3. The register array then performs a right shift, and the next 3 pixels are read from the FPA. When 9 reads have been completed, the array holds the RLPB for the central pixel, which is then sent to the digital coprocessor to compute the histogram. Because the 3×3-pixel windows used to compute the RLBP overlap for adjacent pixels, the next RLBP is completed after 3 reads. The process continues until all pixel values in the region have been read, and the RPG moves to the next region in the image (as shown in Figure 3).

Because the FPA directly outputs the local pixel differences, computing the RLBP requires only a 3×3-bit array instead of the large line buffers that would be required to compute the differences in the RPG. Each RLBP requires 3 reads from the FPA, but because these reads are only used for a 1-bit comparison instead of a complete analog-to-digital conversion, these reads complete significantly faster than when the array operates in conventional mode.

### 4.3. Digital Coprocessor

The digital coprocessor is responsible for computing the histograms of RLBPs from the image, normalizing and centering the data, projecting the resulting histogram vector using LDA, computing the Euclidean distance between the projected vector and a stored database of known faces, and selecting a label for the input image using a nearest neighbor criterion.

**Figure 10:**
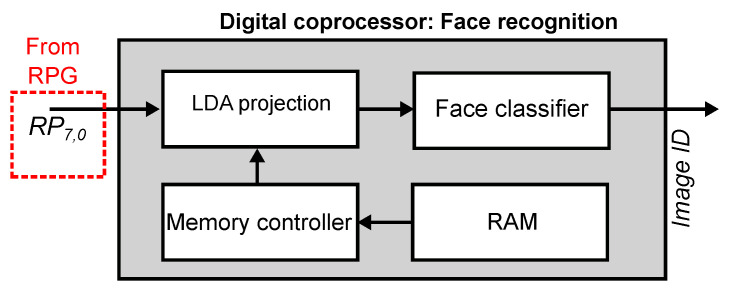
Architecture of the digital coprocessor. The processor receives a stream of RLBPs from the RPG, and simultaneously builds the histogram vector and projects it using LDA. A memory controller retrieves the LDA coefficients from RAM. The Euclidean distance between the projected vector and the contents of database of stored faces is used for classification with the nearest neighbor criterion.

Figure 10 shows the architecture of the face recognition coprocessor. It receives as input the 8-bit RLBP vector RP from the RPG module. The memory controller reads the LDA coefficients from external RAM and sends them to the LDA projection module. This module reads the patterns computed by the RPG for each region of the image, and computes the histogram vector and projects it using the LDA coefficients. Histogram computation and LDA projection are fused into a single step to save memory and arithmetic resources. The output of the LDA module is the feature vector of the input image projected onto the LDA subspace. The face recognition module computes the Euclidean distance between this vector and a set of stored vectors that represent the known faces. The module selects the minimal distance and compares it against a chosen threshold. When the distance is smaller than the threshold, the module outputs the ID of the selected known face. Otherwise, it outputs a null value.

**Figure 11:**
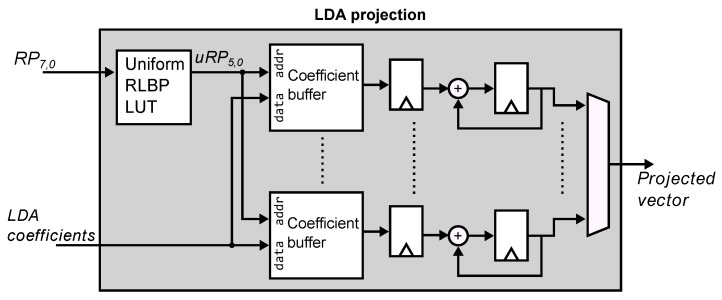
Architecture of the LDA projection module. The module transforms the 8-bit RLBP into a 6-bit uniform RLBP (uRP). For each uRP value received, the module accumulates the value of its corresponding LDA coefficient, thus performing histogram computation and LDA projection in a single step.

Figure 11 shows the architecture of the LDA projection module. The module receives a stream of 8-bit RLBPs from each region of the image. The first step converts the RLBP into a 6-bit uniform RLBP (uRP) using a 256-entry lookup table. In order to avoid the use of multipliers and reduce the amount of local storage required by the LDA projection, the module computes the histogram vector and the multiplication by the projection matrix *W* simultaneously. Each uRP value denotes a position in the histogram vector, for the current region, that must be incremented to build the histogram. The final value stored in this position should then be multiplied by the corresponding set of coefficient values in *W* when the vector is multiplied by the matrix. Instead, every time a new uRP is received, we obtain the value of the coefficient associated with the uRP using a 64-entry coefficient buffer and accumulate the values of the coefficients to directly produce an element of the projected vector. For illustration purposes, let us assume a histogram vector of size 3 and a projection matrix of size 2×3. The traditional projection is computed as shown in Equation (4):

(4) $$y=W^{T}x=\left(\begin{array}{ccc} w_{1,1} & w_{1,2} & w_{1,3}\\ w_{2,1} & w_{2,2} & w_{2,3} \end{array}\right)\cdot \left(\begin{array}{c} x_{1}\\ x_{2}\\ x_{3} \end{array}\right)=\left(\begin{array}{c} w_{1,1}x_{1}+w_{1,2}x_{2}+w_{1,3}x_{3}\\ w_{2,1}x_{1}+w_{2,2}x_{2}+w_{2,3}x_{3} \end{array}\right)$$

where *W* is the LDA projection matrix, *x* is the histogram vector, and *y* is the projected feature vector. Instead, whn the LDA module receives the uRP pattern 1, it retrieves the coefficients $w_{1,1}$ and $w_{2,1}$ from the two coefficient buffers in Figure 11, and accumulates these values in the corresponding registers in the figure. When the module receives the uRP pattern 2, it accumulates the coefficient values $w_{1,2}$ and $w_{2,2}$. If the uRP value is 3, the module adds the values $w_{1,2}$ and $w_{2,2}$ to the registers. When all the uRP values have been read, the registers store the coefficient values of the projected vectors. Thus, for a n×m coefficient matrix, the LDA module requires *n* coefficient buffers of *m* elements. The memory controller block is responsible for reading the coefficient values stored in external RAM storing them in the coefficient buffers.

Projecting the histogram vector *x* with LDA requires normalizing and centering the value of *x*. Because the centering operation is linear, it can be performed in the projected subspace to reduce the arithmetic hardware required to perform the operation, as shown in Equation (5):

(5) $$y=\eta W^{T}\left(x-\mu \right)=\eta W^{T}x-\eta W^{T}\mu ,$$

where $W^{T}$ is the LDA projection matrix, η is the scalar normalization coefficient, and μ is the mean value of the training data vectors. We perform these operations in the projected subspace, locally storing the value of η and the precomputed value of $\eta W^{T}\mu$.

The Euclidean distance between two vectors *p* and *q* can be computed as shown in Equation (6):

(6) $$d\left(p,q\right)=\sqrt{\sum_{i=1}^{N}{\left(p_{i}-q_{i}\right)}^{2}}=\sqrt{\sum p_{i}^{2}-2\sum p_{i}q_{i}+\sum q_{i}^{2}},$$

where d(p,q) represents the Euclidean distance between vectors *p* and *q*, $p_{i}$ and $q_{i}$ are the $i^{th}$ components of vectors *p* and *q*, respectively, and *N* is the dimension of the vectors. Because we are only interested in determining the vector *q* in the database that is closest to the projected input vector *p*, we can use the square of the distance $d{\left(p,q\right)}^{2}$ and avoid computing the square root.

**Figure 12:**
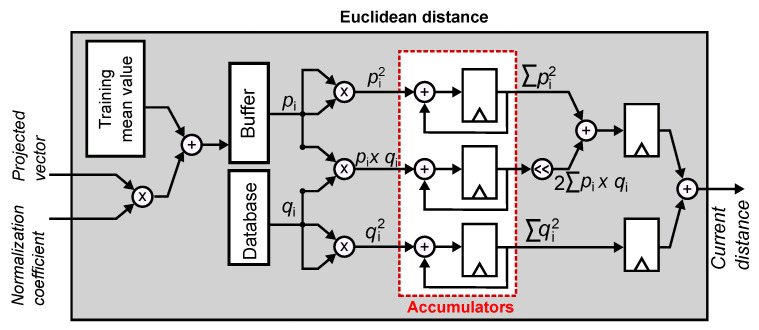
Euclidean distance module. It normalizes and centers the input vector and computes the distance between vectors *p* and *q* as $\sum p_{i}^{2}-2\sum p_{i}q_{i}+\sum q_{i}^{2}$.

Figure 12 shows the architecture of the Euclidean distance module, which computes $\sum p_{i}^{2}-2\sum p_{i}q_{i}+\sum q_{i}^{2}$. The inputs to the module are the projected vector and the LDA normalization coefficient. As described above, the input vector is normalized and centered in the LDA projected space, and stored into a local buffer. Then, the module sequentially computes the distance between the input vector *p* and each vector *q* in the database of known faces. It first computes $p_{i}^{2}$, $p_{i}q_{i}$ and $q_{i}^{2}$, and accumulates their values for 1≤i≤N in three registers. Finally, the values of the registers are added in a two-stage pipeline to compute d(p,q), where the value of $\sum p_{i}q_{i}$ is shifted 1 bit to the left to multiply it by 2. The process is repeated for each know-face vector stored in the local database.

**Figure 13:**
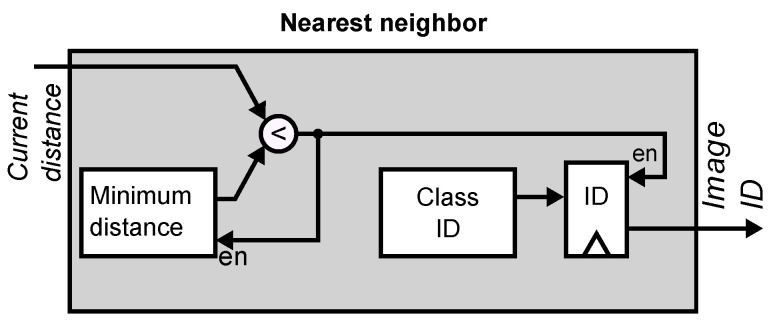
Classification module. The module implements a nearest neighbor criterion by selecting the face label that corresponds to the minimum distance computed between the input image and the stored database of know faces.

Figure 13 shows the final stage of the classifier in the digital coprocessor. The module receives a sequence of distances between the input image and each projected vector stored in the database of known faces. It sequentially computes the minimum value of these distances by comparing the currently-stored distance to the incoming value, and updating the register with the smallest value. Finally, the minimum value is compared to a user-supplied threshold. If the value is smaller than the threshold, the module outputs the face label corresponding to the stored minimum value. Otherwise, it outputs a zero to indicate that the input face is not in the database.

## 5. Results

In this section, we describe the results obtained from a complete system design that comprises the SIS and coprocessor described in Section 4. First, we discuss the physical design of the smart pixel and the implementation of the digital coprocessor on an FPGA. Then, we analyze the classification performance of our proposed method and SIS architecture using a postlayout simulation of the pixel array and the FPGA.

**Figure 14:**
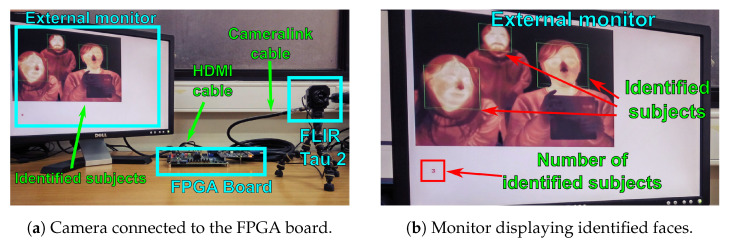
Experimental setup to test the face recognition algorithm. An FPGA board receives IR images from a FLIR Tau 2 camera core and uses a HOG algorithm to detect face locations. The FPGA emulates the smart pixel array and the digital coprocessor. A monitor connected to the FPGA displays the image acquired by the smart pixel array, and the location and number of identified faces. The FPGA sends the labels of the recognized faces to a remote computer via Ethernet.

Figure 14 shows an experimental setup used to validate our face recognition method using an FPGA connected to a thermal IR FLIR Tau 2 camera core. In this case, the FPGA runs a face detection algorithm to locate faces in the acquired image, emulates the smart pixel array that computes the local spatial gradients on the image and the RPG that generates the URLBP values for each pixel, and implements the digital coprocessor. The FPGA outputs the acquired image on an external monitor, and sends out the labels of the recognized faces via an Ethernet link.

### 5.1. Smart Pixel and RPG Implementation

**Figure 15:**
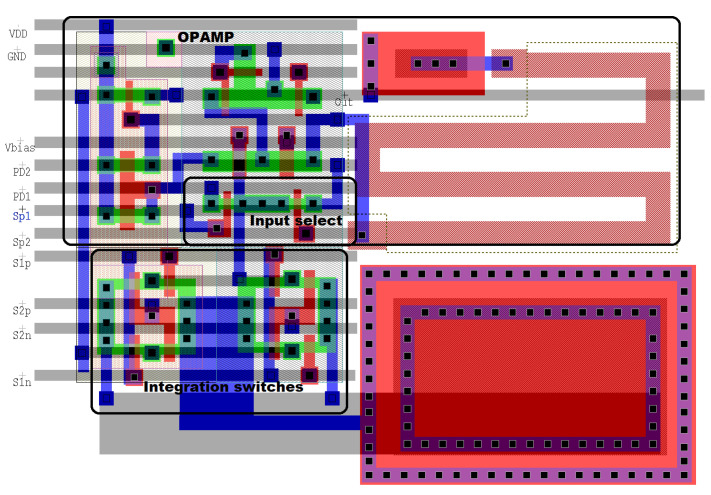
Layout of the smart pixel. We used the design shown in Figure 6, implemented on the TMSC 0.35 μm mixed-signal process. The opamp and integration capacitors are implemented using two poly layers.

Figure 15 shows the layout of the smart-pixel circuit depicted in Figure 6 using the TSMC 0.35 μm mixed-signal process with a 3.3 V supply voltage. We used a poly1-poly2 capacitor for integration, which has a capacitance per area of 950 aF/μm${}^{2}$. Assuming an integration time of 40 μs and a maximum photodetector current of 8nA, the pixel requires a 100 fF integration capacitor of 13.6 μm × 7.7 μm. The dimensions of the complete circuit, including all passive and active elements, are 30 μm × 22.5 μm. Assuming a standard 32 μm × 32 μm pixel [34], the circuit achieves a fill factor of 34%. The extra transistors used to compute local gradients increase the area of the circuit by 26%. Without the switches used to operate in smart mode, the fill factor is 47.6%.

We also ported the design the smart pixel to the 0.18 μm TMSC process, which is more commonly used in the literature [34,35,37]. With a 1.8 V supply voltage and 2 fF/μm${}^{2}$ metal capacitors, the total area of the circuit is 243 μm${}^{2}$, which allows us to achieve a fill factor of 76% in the same 32 μm × 32 μm pixel. In comparison, the integration circuit without the switches for local gradient computation has a fill factor of 79.9%. In summary, our smart pixel is capable of computing spatial differences during integration with a small impact on the fill factor.

Table 1 compares our smart pixels to other designs reported in the literature and discussed in Section 2. Even though the smart pixel described in [34], which computes local differences for edge detection, uses a CTIA only at the column level, it reaches a fill factor of 19%, which is much lower than the 76% reached by our solution in a similar CMOS process. The other designs shown in the table use a much simpler 2-transistor integrator, which is only suitable for capturing images in the visible spectrum. Nevertheless, our design achieves a better fill factor in all cases.

**Figure 16:**
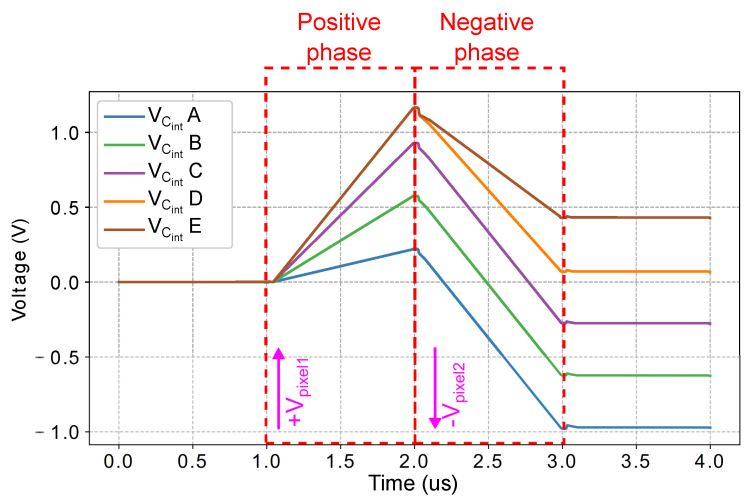
Postlayout simulation of five pixels in the SIS operating in local gradient mode. The graph shows the voltage across the integration capacitor of the CTIA in Figure 7 during the positive and negative integration phases shown in Figure 8.

Figure 16 shows a postlayout simulation of the CTIA during the positive and negative integration phases, as illustrated in Figure 8. The figure depicts the capacitor voltage $V_{Cint}$ for five different pixels in the FPGA. During the positive integration phase, the output voltages increase proportionally to the local photodetector current of the pixel. Then, during the negative phase, the voltage decreases at a rate proportional to the photodetector current of the horizontally adjacent pixel. The voltage after the negative phase is proportional to the difference between the two pixel values. The output voltage $V_{Cint}+V_{bias}$ is then sampled when reading the pixel value.

**Figure 17:**
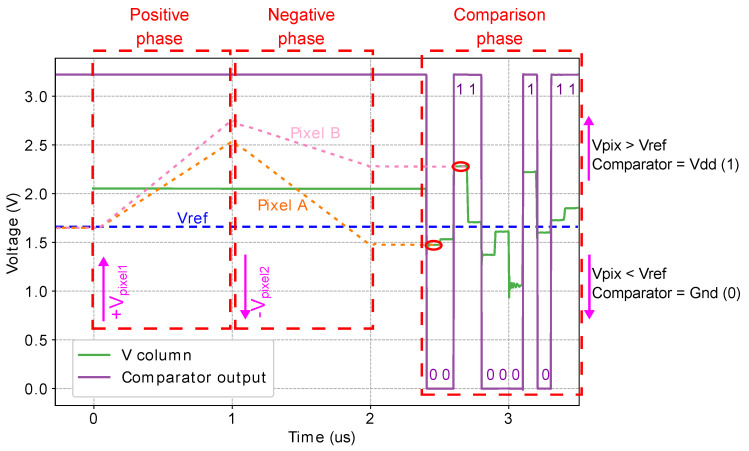
Postlayout simulation of the RPG input comparator while reading multiple pixels. The plot shows the integration phase for the first and third pixel, the voltage input to the comparator, the reference voltage, and the voltage output. Gradient values are read every 50 ns.

The simulation plot in Figure 17 shows the operation of the input comparator of the RPG in Figure 9. During the integration phase, the all pixels compute their local horizontal gradient in parallel. For clarity, Figure 17 shows the output voltage of two pixels (Pixel A and Pixel B). During the comparison phase, the controller performs a row-wise read of the pixel array, sequentially reading the values of three vertically-adjacent cells for each pixel, as described in Section 4.2. The voltage labeled $V_{\mathrm{column}}$ shows the array output voltage, which is the input to the comparator. The output values of Pixel A and Pixel B are the first and third voltages presented to the comparator, respectively, and are sampled at the times circled in red in Figure 17. When the input voltage is higher than $V_{\mathrm{ref}}$, the comparator outputs $V_{\mathrm{dd}}$, or a logic 1. Otherwise, it outputs 0 V, or a logic 0. In the simulation shown in the figure, the comparator outputs the logic sequence 00110001011, which is delivered to the shift registers of the RPG generator to create the RLBP values.

When simulating the operation of an array of 150×80 pixels in the 0.35 μm process, including postlayout parasitics, the readout time of one pixel is 50 ns. Considering that we read each pixel three times in smart mode, the readout time for the complete array is 1.8 ms, which allows us acquire and process images at 556 fps.

### 5.2. FPGA Implementation of the Digital Coprocessor

We modeled the architecture of the digital coprocessor at the register-transfer level using the System Verilog hardware design language, and synthesized the design onto a Xilinx XC7Z020 FPGA. Table 2 shows the resource utilization of our design. The coprocessor uses less than 10% of the logic and distributed memory available on the chip. We used distributed memory to implement the coefficient buffers in Figure 11, because the buffers are small (64 entries), and they need to be accessed in parallel to obtain a new set of coefficients with each uniform RLBP element. The buffer in the Euclidian distance module of Figure 12 uses two embedded RAM blocks, which account for 1.4% of the blocks available on the chip. Finally, the same module uses 6 out of the 220 DSP slices available on the FPGA. This small hardware resource usage leaves ample space on the FPGA to implement additional image processing algorithms. The coprocessor operates at a maximum clock speed of 128 MHz, mostly due to the need to access external memory for the LDA coefficients and the database of stored faces. At this clock rate, the circuit can process $127\times 10^{6}$ pixels per second or one 150×80-pixel image in 94 μs. The power consumption of the circuit operating at this clock frequency, estimated using Xilinx Power Analyzer, is 71 mW.

### 5.3. Method Classification Performance

To test the classification performance of our method, we used four databases: UCHThermalFace database [78], the CBSR NIR face data set [79], the Université Laval Face Motion and Time-Lapse Video Database (UL-FMTV) [80,81], and the Yale Face Database B [82]. Table 3 summarizes the information of spectrum, image size in pixels, number of subjects, number of images per subject, variations in face position, and other conditions of the images in each database.

To evaluate the classification performance of the algorithm on each database, we used 60% of the images for training, that is, to compute the LDA transformation and the stored database of projected faces, and 40% for testing. To reduce overfitting, we used a standard *k*-fold cross-validation technique with 10 iterations. We quantified the performance of the algorithm using the accuracy score, which is defined in Equation (7) for a multiclass classification problem with *N* classes:

(7) $$\mathrm{Accuracy}=\frac{\sum_{i=1}^{N}H_{i}}{\sum_{i=1}^{N}H_{i}+F_{i}}\times 100,$$

where $H_{i}$ and $F_{i}$ are the number of correctly and incorrectly labeled samples of class *i*, respectively. In other words, the accuracy is the percentage of correctly classified images in the test set, computed as the sum of the diagonal elements of the confusion matrix divided by the sum of all the elements in the matrix.

Table 4 shows the accuracy of our method on the four databases, using both RLBP and conventional LBNP to compute the local features. As the table shows, our method performs best with the UCH-TF and CBSR NIR databases, which consist mainly of frontal images with small variations in rotation. Our accuracy is lower, but still above 80%, for YaleFaceB, which is a challenging data set with significant variations in illumination among images. The UL-FMTV contains short video sequences, of which we extracted 24 images for training and 16 for testing, for each subject. The classification accuracy depends largely on which video frames we used to train and test the algorithm: The accuracy is lowest, but still around 75%, when the training and testing frames are far apart in the video frame, mostly due to large variations in rotation angle. When the sets are taken from closer video frames, the algorithm achieves accuracy above 95%.

Table 4 also shows that replacing conventional LBP with our lightweight RLBP descriptor reduces classification accuracy in approximately 2–5%, depending on the database. LBP considers gradients in all directions in a 3×3-pixel window, while RLBP groups only horizontal gradientes in the same window. Therefore, LBP contains more information than RLBP, but we can still capture significant texture information by considering the spatial distribution of horizontal gradientes within a small neighborhood of each pixel. As a result, we can significantly reduce the number of operations at the pixel level with a small loss in accuracy.

Table 5, Table 6 and Table 7 compares the accuracy of our method to other algorithms in the literature using the databases with which they were published. Table 5 reports the algorithms evaluated by Hermosilla et al. [53] using the UCHThermalFace database. Table 6 shows the accuracy achieved with the algorithms reported by Jo et al. [26] with the CBSR NIR database. In all these cases, our method achieves similar or better accuracy than the algorithms reported in the literature. Finally, Table 7 compares the results of our method with YaleFace B database against the algorithms evaluated by Sun et al. [83]. The results show that the accuracy of our algorithm is lower than the reported methods. The main reason for this is that Ahonen’s algorithm does not perform well when there are large variations in illumination between the images in the training and test set. Sun’s algorithm shows more robustness under these conditions, but it requires more computation per pixel. Moreover, this computation can not be easily mapped onto a smart pixel design in the analog domain to exploit pixel-level parallelism in the imager. For NIR and thermal IR images, for which our smart pixel is better tailored, our method delivers better results than the state of the art.

All the experiments described above were executed as a closed-set problem, that is, the test set contains only images of subjects that are also present in the training set. In order to test the performance of our method in an open-set problem, we trained the classifier using only 40 subjects from the UCHThermalFace database, and used a test set with images from all 53 subjects. We used the threshold *THR* described in Algorithm 1 to label the image as unknown if the distance to its nearest is larger than this threshold. In this experiment, using *THR = 8*, the accuracy of LBP+LDA is 95.5%, which is reduced to 93.1% when using RLBP to compute the local features. That is, the accuracy of both methods is reduced by a approximately 3% compared to the closed-set problem.

### 5.4. SIS Classification Performance

**Figure 18:**
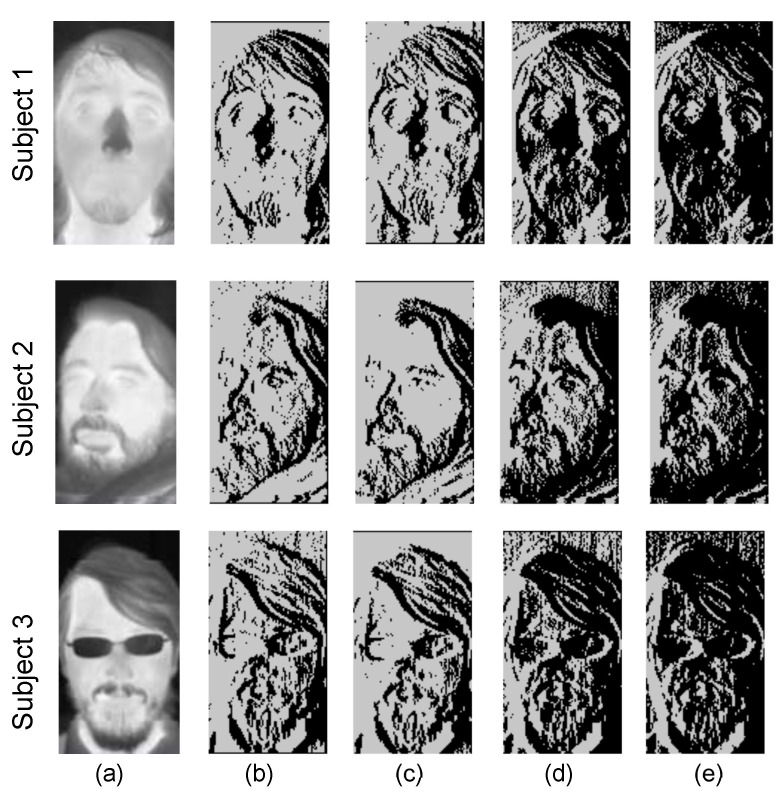
Effect of V${}_{\mathrm{ref}}$ in the RLBP values generated by the RPG for 3 images acquired using a FLIR Tau 2 thermal IR core. (**a**) original IR image, (**b**) RLBP image generated by software, (**c**–**e**) RLBP images generated by the RPG for V${}_{\mathrm{ref}}$ values of 1.665 V, 1.650 V and 1.645 V, respectively.

A circuit parameter that affects classification performance is the reference voltage in the comparator of Figure 9, which computes a digital value for the difference between adjacent pixels. The images in Figure 18 illustrate the effects of $V_{ref}$ in the RLBP values generated by the comparator, for thermal IR images of three different subjects. Figure 18a shows the original image, Figure 18b is the image generated by replacing the pixel values with the RLBP values computed in software, and Figure 18c–e are the RLBP images generated by the hardware setting the value of $V_{ref}$ to 1.665 V, 1.650 V, and 1.645 V.

**Figure 19:**
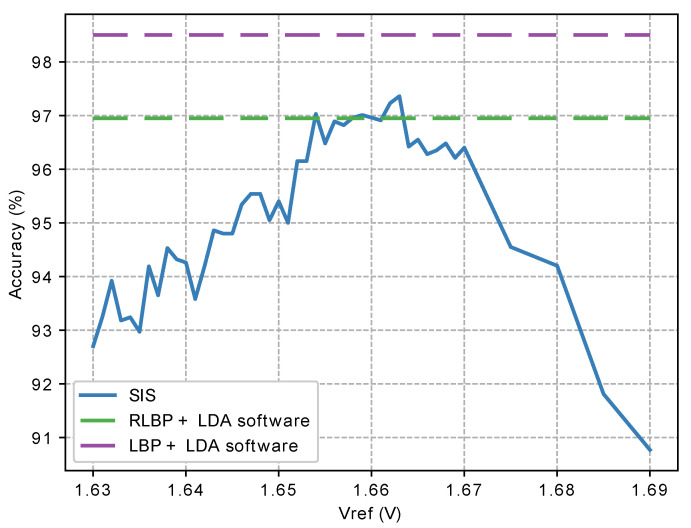
Classification accuracy as a function of the value of the comparator input $V_{ref}$ in Figure 9.

Figure 19 shows the accuracy achieved in our simulations by the complete circuit as a function of $V_{ref}$ using the UCHThermalFace database [78]. For comparison, the figure also shows the classification accuracy achieved by a software implementation of the algorithm, and by the same algorithm in software, using conventional LBP instead of our proposed RLBP. When programmed in software, our algorithm achieves 96.7% accuracy on the test data set, while using LBP achieves 98.5%, but requires 8 times as many comparisons. Varying $V_{ref}$ between 1.63 V and 1.68 V, our hardware implementation using the SIS and digital coprocessor on the FPGA achieves an accuracy above 93%. Setting $V_{ref}$ between 1.655 V and 1.665 V achieves a mean accuracy of 96.5%. These values are slightly higher than the expected value of $V_{ref}=V_{dd}/2=1.65$ V, mostly because of change injection in the feedback and parasitic capacitors of the comparator.

## 6. Conclusions

We have presented a SIS architecture for face recognition that uses local gradients to extract image features based on a lightweight version of LBP. The analog smart pixel sensor computes spatial gradients in the image in parallel during photocurrent integration, and can be configured to output the regular pixel value or the local gradients. A digital coprocessor computes a modified version of Ahonen’s algorithm, where we use LDA to reduce the feature space dimensions and improve class separability.

Postlayout simulations of an array of 150×80 pixels of 32 μm × 32 μm show that the array can deliver up to 556 frames per second. Modifying the integration readout circuit to compute the local gradients allows us to extract local features with a very small impact on fill factor. The digital coprocessor, implemented on a Xilinx XC7Z020 FPGA, can classify a face image in 94 μs, or 10,638 images per second, while consuming 71 mW of power. We use several techniques to reduce on-chip resource utilization, such as storing the LDA coefficients on external memory, and simultaneously building the RLBP histograms and mapping them to the LDA subspace to avoid computing matrix-vector multiplications. As a result, the coprocessor uses less than 10% of the slice LUTs of the FPGA, less than 2% of the on-chip block memory, and less than 3% of the multipliers.

The proposed system has low power consumption and low area utilization, making it suitable for mobile devices and portable systems. Although the CTIA integrator used in the smart pixel is larger than alternative readout circuits, it is suitable for IR and low-light imagers. Computing local differences during photocurrent integration minimizes the impact on circuit area and fill factor, even though by cutting the integration time in half, it may reduce the signal-to-noise ratio of the image sensor in face recognition mode.

When classifying faces using different databases, we observe that our algorithm outperforms other methods in the literature, except when there are large variations in illumination between the training and test data sets. These variations are significantly smaller in IR images, for which our smart pixel has been designed. The results also show that replacing conventional LBP with our proposed RLBP still captures sufficient texture information to perform face classification with a small degradation in accuracy.

We are currently exploring other applications of smart pixels with local difference computation. Specifically, we are exploring using local gradients to correct the fixed-pattern noise present in IR sensors due to nonuniformity in the array. We are also working on other smart pixel designs with local computation for edge detection, image filtering, bad pixel detection and correction, and face detection. We expect that the low resource utilization of the digital coprocessor and the offloading of pixel-level computation to the sensor array will make it possible to integrate multiple algorithms on the same chip or FPGA.

## Figures and Tables

**Table 1:** Comparison of our smart-pixel design to other circuits in the literature.

SIS	Technology	Pixel Pitch μm×μm	Fill Factor	Tested Spectrum	Type of Integrator
Proposed RLBP+LDA face recognition	0.35 μm TMSC	32×32	34%	Visible Thermal IR NIR	CTIA
	0.18 μm TMSC	32×32	76%	Visible Thermal IR NIR	CTIA
Edge detection [34]	0.18 μm 1P4M CMOS	31×31	19%	Visible	2T-integrator, CTIA at column level
LBP Edge detection [35]	0.18 μm CMOS	7.9× 7.9	55%	Visible	2T-integrator
Spatial contrast LBP [77]	0.35 μm	26 × 26	23%	Visible	2T-integrator
4-neighbor LBP [52]	0.35 μm CMOS	64×64	15%	Visible	2T-integrator

**Table 2:** Resource utilization of the digital coprocessor on a Xilinx XC7Z020 FPGA.

	Slice LUTs	Distributed Memory	Block RAMs	DSP Slices
Total used	4345	1298	2	6
Available	53,200	17,400	140	220
Percentage	8.2%	7.6%	1.4%	2.7%

**Table 3:** Databases used to test the performance of the proposed method.

Database	Spectrum	Image Size (Pixels)	Number of Subjects	Images per Subject	Face Positions and Conditions
UCH-TF	Thermal IR	150×81	53	28	Rotations and expressions
CBSR NIR	Near IR	640×511	197	20	Frontal, with and without glasses
UL-FMTV	Thermal IR	128×128	238	Short video sequence	Rotations
YaleFace B	Visible range	192×168	38	64	Frontal, expressions and light variations

**Table 4:** Accuracy of our method with RLBP and LBP using different databases.

	UCH-TF	CBSR NIR	UL-FMTV	YaleFaceB
RLBP+LDA	96.7%	96.0%	75–95.9%	76.4%
LBP+LDA	98.5%	98.1%	79–97.1%	82.9%

**Table 5:** Accuracy of our RLBP+LDA and LBP+LDA methods compared to other face classification algorithms discussed in [53], using the UCH-Thermalface [78] database.

Method	RLBP+LDA	LBP+LDA	GJD	WLD	LBP
Accuracy	96.7%	98.5%	96.6%	94.4%	92.0%

**Table 6:** Accuracy of our RLBP+LDA and LBP+LDA methods compared to other face classification algorithms discussed in [26], using the CBSR NIR [79] database.

Method	RLBP+LDA	LBP+LDA	NIRFaceNet+Aug	NIRFaceNet	FaceNet
Accuracy	96.0%	98.2%	96.6%	94.8%	84.1%

**Table 7:** Accuracy of our RLBP+LDA and LBP+LDA methods compared to other face classification algorithms discussed in [83], using the Yale face database B [82].

Method	RLBP+LDA*	LBP+LDA*	Sun’s Kernel	CRC	ELM	Tanh
Accuracy	76.4%	82.9%	98.33%	96.82%	96.44%	96.34%

## Data Availability

This study use the following publicly available datasets: Extended Yale Face Database B (http://vision.ucsd.edu/~leekc/ExtYaleDatabase/ExtYaleB.html (accessed on 24 March 2021)), CBSR NIR Face Dataset (http://vcipl-okstate.org/pbvs/bench/Data/07/download.html (accessed on 24 March 2021)), and The Université Laval Face Motion and Time-Lapse Video Database http://www.qirt.org/liens/FMTV.htm (accessed on 24 March 2021). The UCH-ThermalFace dataset can be obtained from the authors of reference [78].
